# Traumatic brain injury among older adults: urgent need for implementation of specific rehabilitation needs and tailored to low-middle-context

**DOI:** 10.3389/fnhum.2026.1809177

**Published:** 2026-05-01

**Authors:** Franklin Chu Buh, Harry Mee, Fahim Anwar, Gilbert Mua Akwa, Ignatius Esene, Peter J. A. Hutchinson

**Affiliations:** 1 NIHR Global Health Research Group on Acquired Brain and Spine Injury, University of Cambridge, Cambridge, United Kingdom; 2 Faculty of Medicine and Pharmaceutical Sciences, Department of Physiotherapy and Physical Medicine, University of Dschang, Dschang, Cameroon; 3 Department of Rehabilitation Medicine, Addenbrooke's Hospital, Cambridge University Hospitals NHS Foundation Trust, Cambridge, United Kingdom; 4 Department of Public Health, Liverpool John Moores University, Liverpool, United Kingdom; 5 Faculty of Health Sciences, Department of Neurosurgery, University of Bamenda, Bamenda, Cameroon; 6 Department of Clinical Neurosciences, University of Cambridge, Cambridge, United Kingdom

**Keywords:** consensus, low-middle-income countries, management, neurorehabilitation, older adults, traumatic brain injury

## Introduction

Traumatic brain injury (TBI) is a silent global threat (Alkhaibary et al., 2021), annually affecting about 69 million individuals worldwide (Dewan et al., 2018; Naik et al., 2023). It is a leading cause of death and disability (Capizzi et al., 2020; Wongchareon et al., 2020; National Academies of Sciences Engineering Medicine: NASEM, 2022), with low-and middle-income countries (LMICs) bearing a disproportionate TBI burden with significant consequences for affected individuals, families and health systems (Rahman et al., 2025; Buh et al., 2023a). Although the definition of older adults remain subjective, current guidelines classify all TBI patients aged 65 and older as having geriatric TBI (Menon, 2024). The magnitude of the problem is growing, and it is projected that the aging population trends observed in high-income countries are extending to LMICs (Menon, 2024). This means that the global public health burden of TBI in older adults will rise dramatically but disproportionately for LMICs in the years ahead (Menon, 2024). Studies by Buh et al. (2023a) in Cameroon revealed a prevalence of 7.3% for TBI in adults >60 years (Buh et al., 2022) and 4.4% in adults aged >65 years (Buh et al., 2023a). Furthermore, a recent study reported an extremely high incidence of TBI in the Southwest region of Cameroon of 975.57 per 100, 000 population (Embolo et al., 2021), which is significantly greater than previous findings (Dewan et al., 2018). This increasing incidence of TBI in LMICs likely informs of an increase in TBI in the elderly population, that already has several other comorbidities (Lagares et al., 2025). Furthermore, TBI-related mortality in LMICs is 3-fold to 4-fold higher than that reported in high-income countries (Naik et al., 2023). Faced with this rising incidence and burden of TBI in the elderly, Lagares et al. (2025), highlighted several uncertainties to consider when treating of TBI in older adults compared to younger adults: assessment, diagnosis and management.

In persons ≥ 65 years, TBI is responsible for more than 80,000 emergency department visits each year, of which three-quarters result in hospitalization. Adults ≥ 75 years have the highest rates of TBI-related hospitalization and death (Thompson et al., 2006). Falls are the leading cause of TBI for older adults in high income countries, while for LMICs, falls and road traffic crashes battle for the main cause of TBI in older adults (Buh et al., 2023b, 2022, 2023a). More than 25% of older adults fall each year, with TBI being one of the most common injuries encountered in older adults after a fall (Colón-Emeric et al., 2024).

Considering the increasing complexity of managing TBI in older adults and problems specific to LMICs like limited resources, inadequate infrastructure, and a shortage of trained healthcare professionals, rehabilitation of older adults with TBI is a critical issue that needs to be addressed urgently. In LMICs, TBI rehabilitation faces many challenges, reflecting a high disparity rate as compared to high-income-countries (HICs) (Buh et al., 2023b). Specificities in the Rehabilitation of TBI in older adults are not debated in most LMICs. As of now, there is no consensus for the rehabilitation of TBI in most LMICs, let alone specific rehabilitation aspects to consider for older adult TBI patients. Therefore, there is an urgent need for a reflection on the specific rehabilitation needs for TBI in older adults globally and regionally tailored (HICs and LMICs). Thus, this opinion paper seeks to call attention to the need to understand the trends of TBI in older adults and address specific rehabilitation needs.

## Management and rehabilitation of TBI in older adults

Rehabilitation after moderate-to-severe traumatic brain injury (TBI) is a complex multidisciplinary process that requires a clear understanding of the different causes and effects of the injury, pre-existing comorbidities and frailty (Shen et al., 2025). Older adult TBI is different from TBI in younger people and therefore deserves an age-appropriate approach to management and rehabilitation. Pre-existing conditions, frailty, and disability are common and may confound the initial evaluation and diagnosis of TBI. Older adults with existing comorbidities are often excluded from several reviews, and this might hinder the development of age-specific considerations in their management, due to a lack of scientific evidence (Albrecht and Gardner, 2023). These shortcomings place a blindside in the specific considerations for TBI management in older adults, which could be detrimental to these individuals, families, health systems and governments at large. Furthermore, recent estimates project significant increase in the population of older adults, with higher risk of falls causing TBI (Menon, 2024). Meanwhile the above mentioned studies (Shen et al., 2025; Albrecht and Gardner, 2023) agree that special considerations like pre-existing comorbidities and frailty should be specifically considered when treatment TBI in older adults, they did not address specific rehabilitation considerations for TBI in older adults constituting a significant gap. According to a recent Delphi study by Lagares et al. (2025), involving 72 experts from across the world: Europe, the United States, Latin America, Africa, and Asia, responded to 62 statements. The consensus areas in the specific management of TBI in older adults are: definition of old age, which should include age, frailty, level of independence, comorbidities; patient's assessment, which should include frailty assessment with a more specific frailty tool, drugs, and neurosurgical consultation; CT scans which should be recommended early for all older adult patients with TBI; Biomarkers, where a clear guideline for the use of biomarkers in predicting CT scanning should be developed for elderly TBI patients. Other areas of agreement included surgical care, stopping antithrombotic treatment, ICU management, where targets were aligned with those of younger TBI patients, discharge, and rehabilitation. Although there was an agreement for physical and cognitive rehabilitation after TBI in elderly patients, there was no consensus on the rehabilitation needs and specificities for older adults with TBI. Rehabilitation experts (Rehabilitation medicine physicians, physiotherapists, occupational therapists) were not part of this consensus, as experts who participated were mainly of neurosurgical, emergency and intensive care. Thus, the consensus did not address a major problem which is neurorehabilitation of TBI in older adults. The former represents a significant gap that needs to urgently be addressed for the long-term management of TBI in older adults and improved treatment outcomes. In addition, there is a need for a global consensus on the management and neurorehabilitation of TBI in older adults and adaptation to limited resources in LMICs, including more experts like rehabilitation medicine physicians, neuro-physiotherapists, and policy makers. An important management area is the prevention of repeated falls and repeated TBI in older adults presenting with TBI. Previous TBI is a known risk factor for repeat TBI, particularly among older adults, who may have pre-existing risk factors for falls and in whom the TBI itself may produce new post-concussive symptoms that will exacerbate fall risk (Lagares et al., 2025). If this downward spiral is not adequately addressed, TBI in older adults will continue to pose a significant burden to society at large and most particularly in LMICs with consequential shortages of resources.

## Overlap in management recommendations between HICs and LMICs

As the world Health Organization (WHO) predicts an increase in the burden of TBI, LMICs are set to have a disproportionately heightened burden, accounting for over 90% of all trauma-related fatalities and have TBI rates which are three times higher than in HICs (Dulf et al., 2021; Griswold et al., 2024). Despite this significant overlap in the burden of TBI between LMICs and HICs, existing TBI management guidelines are primarily designed for HICs with advanced medical infrastructure and sufficiently available multidisciplinary teams (Best Practice Guidelines, 2024). These resources are tremendously limited in LMICs, leading to suboptimal outcomes when these recommendations are implemented in these settings (Griswold et al., 2024). Aside the lack of infrastructural and financial resources, LMICs are also faced with weak public health systems, challenges in building resilient health systems and governance and leadership problems. All these added to workforce crises distinctly challenging in LMICs (Phelan et al., 2022; Kodali, 2023). Although healthcare specialists may be well trained in LMICs, however, there is insufficient workforce, lack of proper equipment, limited hospitals outside major cities, lack of specialized personnel in rural areas and lack of appropriate rehabilitation services (Dulf et al., 2021). Therefore, recommendations for the management of TBI made for HICs may not be directly applicable in a low-middle-income context without specific adaptations. For example, prehospital care for TBI markedly influences outcome from traumatic brain injury through all ages (Hawryluk et al., 2023), but the recommendations concerning pre-hospital care for TBI are highly unrealistic for most LMICs. The WHO and other guideless recommend by-stander care, medical transportation, intubation, monitoring, ventilation, fluids administration, alerting the emergency services where patients are transported before arrival, among others (Chowdhury et al., 2014; Hawryluk et al., 2023). LMICs still face significant challenges in implementing such recommendations, as over 90% of brain injured patients are transported to the hospitals through non-medical means, and so receive no pre-hospital care (Buh et al., 2023b, 2022). Another important aspect of management of TBI is prevention, where major disparities occur between high- and low-income settings. For instance, the main causes of TBI in LMICs are road traffic accidents, while for HICs, falls account for most of TBI across all ages (Maas et al., 2022; Buh et al., 2023b, 2022). Therefore, preventive strategies for TBI will not be the same between LMICs and HICs. Thus, it is crucial and urgent to consider the present context of LMICs and bring fought recommendations that can relate in limited resource settings. If this is not addressed, LMICs run the risk of posing a significant hindrance to reducing the global burden of traumatic brain injury. Meanwhile age-appropriate management initiatives for TBI are globally encouraged, the need to develop and implement regionally adapted recommendations for the management and rehabilitation of TBI in older adults is urgent.

Another major area of concern around disparities in TBI management in older adults between HICs and LMICs is lack of health coverage. Most of the populations of LMICs finance injuries and other health conditions through out-of-pocket payments (Nanda and Sharma, 2023; Buh et al., 2023a; Thakur and Faizan, 2023). The study of Arora et al. (2026) highlights that out-of-pocket expenditure in TBI patients significantly impacts the spending on daily necessities—food, housing, and education and compound catastrophic health expenditure. According to the World Bank Group, 44% of the global population live in poverty among which a greater proportion are in LMICs. In 2024 for example, Sub Saharan Africa, accounting 16% of the world's population, 67% of people living in extreme poverty (World Bank Group, 2024). This situation is crucial as people in LMICs struggle to deal with everyday life needs making TBI care much more challenging. Furthermore, poverty, disability and older age are associated (Banks et al., 2017), which add-up to out-of-pocket care of TBI makes the equation more complex to resolve. LMICs need to be informed on alternative strategies to address the context-specific burdens posed by TBIs in older adults.

## Why should older adults with traumatic brain injury be considered differently?

Management and rehabilitation of TBI in older adults must consider comorbidities and frailty, the treatment of pre-existing disorders, the reduced potential for recovery, the likelihood of cognitive decline, and the avoidance of future falls (Depreitere et al., 2025). Older adults with TBI experience higher morbidity and mortality and slower recovery trajectories. They have worse functional, cognitive, and psychosocial outcomes months or years post-injury than do younger patients (Gardner et al., 2018). Despite the growing reports and scientific convergence on the fact that frailty assessment should constitute a major part in the management of TBI in older adults, there is still limited evidence linking frailty and worst TBI outcomes in older adults. However, the study of (Gerber et al. 2023) reiterates that a greater understanding of frailty in the context of older TBI would improve outcomes and prognostication. Furthermore, Jonsson et al. (2025) interestingly, in a small-scale study found that older TBI adults with higher clinical frailty scores had higher mortality rates 30 days after TBI than those with low frailty scores. Such findings, therefore, underscore the urgency in early frailty integration in the management and rehabilitation of TBI in older adults. In another review, frailty was considered as an independent predictor of length of hospital stay and recovery status among older adults with traumatic brain injury (Cray et al., 2025).

Prognostication of TBI in older adults remains highly uncertain, as the IMPACT and CRASH TBI statistical models included patients with a mean age in the 30s (Best Practice Guidelines, 2024). Multiple medical comorbidities and cognitive impairment frequently precede TBI among older adults and likely complicate management and expected rehabilitation outcomes (Dams-O'Connor et al., 2016).

Another area of specificity is the evaluation of pupillary response, which may be confounded by preexisting chronic ophthalmic diseases (e.g., glaucoma or cataract disease). In addition, the use of anticoagulants in many older patients may complicate the imaging recommendations. In patients who are anticoagulated, the incidence of intracranial hemorrhage (ICH) is higher, thus, more liberal use of early CT scanning may be appropriate (Best Practice Guidelines, 2024).

According to a report by Menon (2024), several factors exacerbate the severity of TBI in older adults, such as the dura adherence, brain atrophy, decreased compliance, diminished neuronal plasticity, increased risk of cerebral microbleeds, and impaired cerebral autoregulation. Another concern raised by Menon, is the application of general adult TBI guidelines for the elderly, particularly those with comorbid conditions. It should be noted that medical and rehabilitation treatments are inextricably linked to each other. Thus, understanding the specificities of TBI in older adults inform adequate rehabilitative strategies. There is an urgent need to agree on and implement a consensus on the management and rehabilitation of TBI in older adults.

To date, no consensus has been reached regarding the neurorehabilitation of TBI in older adults. The absence of specific rehabilitation recommendations for older adults highlights the need for stronger evidence to enhance prognostication and improve outcomes. This comes at a time when the world still faces consequential barriers to implementing the WHO's Rehabilitation 2030 initiative, highlighting the profound unmet need for rehabilitation worldwide and the importance of strengthening health systems to provide rehabilitation. The initiative emphasizes that rehabilitation should be available for the entire population and throughout all stages of the life course, including older adults (World Health Organisation, 2025).

## What makes this opinion unique and relevant?

This is opinion paper is unique in the following aspects:

- It brings out the overlap in the management and neurorehabilitation of TBI in older adults. Previous works have mainly addressed management of TBI in older adults with no overview on the context-specific aspects of the matter.
- It also highlights the need for a consensus for rehabilitation care specific for older adults with TBI. To the best of our knowledge, this is the first paper to strongly recommend a consensus on the rehabilitation of TBI in older adults for LMICs
- This opinion also highlights the specificities of to be considered in the rehabilitation of TBI in older adults in a comprehensive 6 aspects outlined in a subsequent section below
- It highlights the different phases of neurorehabilitation of older TBI patients and treatment modalities per phase tailored to limited resource context
- This opinion also proposes setting up context-specific rehabilitation teams like transdisciplinary teams which appear to be cost-effective than the standard multidisciplinary team
- The opinion above all proposes in Table 1 10 key recommendations and explanations to improve rehabilitation outcomes in older TBI patients.

**Table 1:** Key recommendations to consider in the rehabilitation of older adult's TBI in LMICs.

No	Recommendations and explanations
**1**	Setting up transdisciplinary neurorehabilitation teams (TDT) that could consist of neurologists, neurosurgeons, geriatric physicians, physiotherapists, Occupational Therapists, Speech and Language Therapists, psychiatrists, and clinical psychologists. TDTs are cost effective, and suitable for resource limited settings (Maas et al., 2022). This is indispensable for effective rehabilitation of older adults with TBI. TBI in older age is a very complex situation that would need to benefit from a holistic and integrated care by breaking down professional silos, enabling more efficient service provision necessary for better overall outcomes.
**2**	Compulsory assessment of older TBI adults by neurorehabilitation teams considering specificities in hyperacute (ICU) and acute stages (hospitalization). Specific assessment of comorbidities (like heart disease, stroke, dementia, chronic respiratory disease, musculoskeletal problems like arthritis/osteoarthritis etc.), frailty, pre-existing conditions and treatment receiving, cognitive problems like delirium, needs to be systematically implemented.
**3**	In hyperacute care, added to previous the recommendations on neurorehabilitation of TBI by Buh et al. (2023a), special care should be taken when mobilizing older patients with TBI in stable ICP, to prevent fractures resulting from mobilization stress. Older age is associated with decline in bone density and increase likelihood of fractures (LeBoff et al., 2022). Early mobilizations remain a cornerstone to preventing delirium, reducing stress, contractions, deformation, and improving respiratory health and rehabilitation outcomes.
**4**	In acute care (hospitalization or neurosurgical ward): intensification of education for patients, and families or caregivers on age-related recovery, communication and safety, cognitive and emotional changes, burnout prevention, and care chain (Hart et al., 2018), thoracic expansion and mobilization exercises; active assisted range of motion to active range of motion of the upper and lower limbs depending on the cognitive state of the patients; static stretching of the hamstrings, quadriceps; isometric contractions of the quadriceps, gluteal muscles, hamstrings, anterior and posterior lodge muscles of the leg, biceps and triceps; bed mobility; ambulation out of bed with a wheelchair. Psychologic or psychiatric intervention where applicable.
**5**	In the subacute phase, older TBI patients are usually discharged to neurorehabilitation centers or physiotherapy services. Here, a more comprehensive and detailed assessment is done by neurorehabilitation teams to determine the individualized needs for rehabilitation, and which professionals need to intervene. At this stage, intensive physical and cognitive rehabilitation should be offered respectively for TBI patients with physical and cognitive disabilities. Frailty examination and fall risk assessment needs to be conducted. Fall risk assessment must consider intrinsic, socio-cultural, and environmental factors. Finally, the implementation of inclusive fall prevention programs is capital at this stage of rehabilitation of older adults with TBI achieved through mitigation of identified fall risks and physical interventions like sub-symptom strength and balance exercises.
**6**	Post-acute phase or chronic phase where patients are within their communities. Community-based rehabilitation programs (CBR) should be established and implemented with the primary objective of reintegrating TBI survivors into the community as much as possible. Such programs exist in high income settings but still limited or lacking in most low-middle-income countries, where the burden of traumatic brain injury in older adults is paradoxically many folds higher. Implementation of CBR programs is crucial to reducing disabilities linked to TBI in older adults, and in improving quality of life and cognitive health.
**7**	Caregiver-directed programmes (CDP) and NGO-based programmes: The CDP is a good concept to integrate in the rehabilitation of TBI in older adults. It empowers family and friends to be directly involved in providing education, skills, managing behavioral and social changes, facilitating their community living, and reaching out to brain or injury support organizations, for assistance (Brain Injury Association of America, 2026). NGO-based programs help in building resilience and connecting survivors and their families through community groups and rehabilitation. Brain injury NGOs in HICs can empower local NGOs in LMICs, through training and funding opportunities. TBI survivors who have successfully integrated into the community should be used to build resilience and encourage older adults with TBI that improvement is possible.
**8**	Improving policy. National and international health systems should develop and implement policies that consider older patients as specific entities in the care of traumatic brain injury. Also, they should drive policies to develop and fund rehabilitation services across urban and rural settings and implement WHO fall prevention programmes and WHO rehabilitation 2030 initiative. National and International non-governmental organizations should expand their involvement in rehabilitation initiatives of the elderly especially in LMICs where these are not part of national priorities. Rural areas in LMICs often have higher proportion of older adults and lack adequate health facilities for the care for TBI. Effectively decentralizing healthcare in LMICs, this will not only bring more infrastructural growth to these areas but also improve management and rehabilitation outcomes for older adults.
**9**	Consensus for rehabilitation of TBI in older adults. Although there exists a consensus for the management of TBI in older adult (Lagares et al., 2025), it is not known if it has widely been accepted or implemented. Furthermore, the consensus did not address specifically rehabilitation of these patients. National, international, and global health stakeholders, interdisciplinary health experts including clinical and rehabilitation professionals needs to develop consensus that should be implemented and adaptable in high and low- or middle-income context.
**10**	Training of rehabilitation and clinical professionals is needed to spearhead the management of traumatic brain injury in older adults which remain a complex and delicate process. This training should focus more on LMICs where there is tremendous lack of human, financial and infrastructural resources.

## What is the way forward?

Research suggests that early onset of medical and neuro-rehabilitative care in the trauma center and more intensive neurorehabilitation in the rehabilitation environment help to aid recovery and improve functional outcomes in older adults with TBI (Buh et al., 2023a). According to the Best Practice Guidelines (Best Practice Guidelines, 2024), early multidisciplinary rehabilitation is safe, feasible, and cost-effective for patients with TBI who have not yet achieved medical stability and/or continue to require mechanical ventilation. It mitigates complications of immobility, critical illness, delirium, and cognitive and psychological disorders. Although LMICs possess significant financial, infrastructural and human resources limitations to implement effective management of TBI in older adults, these measures could be adopted if attention is paid to this growing threat. Due to insufficient human resources in most LMICs, the development of multidisciplinary teams and implementation of neurorehabilitation programs as seen in high-income countries, may not be feasible. However, transdisciplinary neurorehabilitation teams composed of physicians (Neurologists, neurosurgeons, geriatric medicine specialists, Rehabilitation medicine physicians), physiotherapists, occupational therapists, Speech and Language therapists, clinical psychologists, and psychiatrists, should be implemented. Furthermore, transdisciplinary teams have been reported as the gold standard for neurorehabilitation teams especially in resource limited settings (Karol, 2014). Transdisciplinary goals for rehabilitation of Traumatic brain injury in the elderly are summarized in Table 2.

**Table 2:** Proposed transdisciplinary goals for rehabilitation of traumatic brain injury in the elderly.

No	Goals
1.	Serial assessment of consciousness, Frailty and comorbidities
2.	Posture and tone management during the period of hyperacute and acute care including the use of orthotics or splints
3.	Early mobilization interventions, prevention of bedsores, limitation of bed rest and delirium prevention
4.	Improvement of sleep quality
5.	Early assessment of communication (and swallowing, if appropriate) and provision of assistive technology
6.	Plan for long-term nutrition
7.	Assessment of ongoing rehabilitation needs, liaising, and transfer to a long-term rehabilitation facility
8.	Fall prevention programs implementation (to avoid repeat TBIs which are deadly in elderly population)
9.	Assessment of mental capacity and facilitating best interest decisions
10.	Prognostication
11.	Effective education and family support

The acute care rehabilitation plan should be developed in consultation with a multidisciplinary team including physical and rehabilitation medicine physicians, neuro-physiotherapists, clinical psychologists, and geriatric medicine specialists among others (Buh et al., 2023a; Best Practice Guidelines, 2024).

Early mobilization mentioned in Table 2 above, is defined as initiation of mobilization activities within the first 48 h of injury, or as soon as neurosurgical, hemodynamic, and respiratory stability are achieved. A multitude of evidence demonstrates the efficacy of early mobilization in critically ill patients. Immobilization has been known to give rise to long-term detrimental effects, such as muscle breakdown, bone resorption, cardiac and pulmonary complications, deep vein thrombosis, pulmonary embolus, and long-term cognitive and physical impairments, which will lead to a longer length of stay (Karol, 2014). Considering that older adults already experience a decline in cardiovascular activity and musculoskeletal fitness, the effects of immobilization after TBI are tremendous compared to those in younger patients. Therefore, early mobilization is extremely important to consider in the rehabilitation of these patients. Furthermore, early mobilization is known to reduce the incidence of delirium among elderly TBI patients, as well as improve strength, functional recovery, quality of life, and earlier ventilator liberation (Nazwar et al., 2023; Zang et al., 2020).

## Specificities in the rehabilitation of TBI in older adults

Management and rehabilitation in older adults must consider:

1. Comorbidities/Baseline before injury and frailty (F-R-A-I-L- scale),
2. The treatment of pre-existing disorders,
3. The reduced potential for recovery,
4. The likelihood of cognitive decline,
5. The avoidance of future falls, and the
6. Lack of specific prediction models in older patients

**What to consider LMIC context?**

**Setting up transdisciplinary neurorehabilitation teams:** physicians, physiotherapists, Occupational therapists, psychiatrists, and clinical psychologists, rehabilitation physicians (where applicable) as they are fewer in most LMICs particularly in Africa (Tannor et al., 2022). Assessments of older TBI patients by the neurorehabilitation teams at the hyperacute stage (ICU) and acute stage.

**Fall risk assessment:** Time up and go test (TUG), Tinetti Performance Oriented Mobility Assessment (POMA), Tandem Stand (World Health Organization, 2008).

## Neurorehabilitation phases to consider in older TBI patients

The phases of neurorehabilitation after traumatic brain injury are the same for older adults as in younger adults: Phase I (hyperacute and acute), Phase II (Subacute), and Phase III (post-acute). These phases are detailed in our previous publication (Buh et al., 2023a).

In Phase I (ICU), where there is unstable intracranial pressure, education of family or caregivers, Neuromuscular electrical stimulation of the lower limbs is necessary to ensure proper circulation and prevent muscle wasting and retractions of soft tissues.

Where intracranial pressure is stabilized: regular changing of positions, postural drainage, manual chest vibrations and percussion, passive joint mobilization, chest proprioceptive neuromuscular facilitation). Care must be taken when mobilizing these patients because their skeletal systems are fragile and may be prone to fractures.

At hospitalization, Thoracic expansion, and thoracic mobilization exercises, active assisted range of motion to active range of motion of the upper and lower limbs, static stretching, isometric contractions, bed mobility ambulation out of bed with a wheelchair, psychological or psychiatric intervention, depending on the patient's cognitive behavior.

In phase II (Rehabilitation centers), where intensive physical, psychological and cognitive rehabilitation is observed.

Phase III (Community). To date, community rehabilitation services for traumatic brain injuries are absent in many LMICs. There is an urgent need for community-based programs with a focus on enabling TBI patients to reintegrate the community as much as possible.

## Frailty assessment and fall prevention

Frailty is an independent predictor of length of hospital stay in older adults with TBI, discharge disposition, and functional recovery in older adults with TBI. Frailty scales specific to traumatic brain injury in the elderly need to be developed and implemented (Colón-Emeric et al., 2024). According to the WHO guidelines on fall prevention, fall risk is highest in low-income settings (World Health Organization, 2008). Falls impose a disproportionately greater burden in LMICs than in HICs. In addition, education on safe mobility practices and home modifications, such as improving lighting, eliminating tripping hazards, or installing grab bars, can significantly reduce fall risks. According to the Lancet Neurology (The Lancet Neurology, 2025), a fall prevention strategy could reduce the incidence of injury among older adults. In multidisciplinary team management, these interventions can be even more effective, as they incorporate the contributions of physical therapists, nurses, and social workers (Wongchareon et al., 2020). Generally, the WHO groups fall-prevention interventions at three levels: (i) Policy level, (ii) prevention, (iii) practice-interventions. Considering the policy level, the WHO advocates that to effectively manage falls in an aging society, healthy and strategic public policies are needed to provide a tailored vision, set priorities and develop institutional standards. This should involve leadership, collaboration, and education. Alongside good leadership and collaboration, education is an essential strategy for building the necessary capacity for effective fall prevention policy and practice. Prevention is the second level of fall management in older adults according to the WHO. It is implemented at three levels: first, in the community where health and environment risk-factor assessment, with interventions based on assessment results, is highly effective in reducing falls among community-dwelling older persons who are cognitively okay. Secondly, residential settings where strategies like gait training, exercise programs, nutritional support, and environmental modifications are important (Best Practice Guidelines, 2024; Zang et al., 2020).

## Summary points of key recommendations

- Setting up transdisciplinary neurorehabilitation teams are cost effective for LMICs with the available professionals.
- Compulsory assessment of older TBI adults by neurorehabilitation teams considering specificities of older TBI adults in hyperacute (ICU) and acute stages (hospitalization).
- Effective implementation of management and rehabilitation strategies across major rehabilitation pathways: hyperacute and acute, subacute and post-acute pathways.
- National and international health systems should develop and implement policies that consider older patients as specific entities in the care of traumatic brain injury. They should also drive policies to develop and fund rehabilitation services across urban and rural settings, to meet with the WHO's rehabilitation 2030 initiative.
- National, international, and global health stakeholders, interdisciplinary health experts including clinical and rehabilitation professionals needs to develop consensus that should be implemented and adaptable in high and low- or middle-income context.
- Global and national initiatives need to promote training of rehabilitation and clinical professionals in LMICs, starting with professionals that are severely deficient, but crucial for the management and rehabilitation of TBI in older adults. These include rehabilitation professionals like rehabilitation physicians, neuro-physiotherapists, neuro-nurses, occupational therapists, and clinical professionals like neurosurgeons, intensive care specialists.
- It is imperative that LMICs decentralize healthcare facilities capable of managing traumatic brain injury. As of now, a large majority of health facilities with technical knowhow to manage TBI are in major cities and lacking in rural areas where the older population reside the most. This will help to reduce burden and improve outcomes.

## Conclusion

Management and rehabilitation of traumatic brain injury in older adults needs to consider several specificities to avoid case fatalities. Comorbidities, treatment received for pre-existing conditions, frailty, and fall risk assessments are crucial for this group of patients. Rehabilitation must consider the multi-system decline observed in older patients with TBI. Fall prevention programs needs to be implemented and inspired by intrinsic, social and environmental fall risk factors. National, international and global health systems must work to development and implement a regional-tailored consensus for the rehabilitation of older adults with TBI.
